# Genomic prediction for stem rust resistance in the southern United States elite oat (*Avena sativa* L.) germplasm

**DOI:** 10.3389/fpls.2026.1795871

**Published:** 2026-03-19

**Authors:** Janam Prabhat Acharya, Naeem Khan, Sudip Kunwar, Jordan McBreen, Samuel A. Adewale, Stephen Harrison, Ellen Melson, Daniel Hathcoat, Raja Sekhar Nandety, Jason Fiedler, Md Ali Babar, Kathy Esvelt Klos

**Affiliations:** 1 Department of Agronomy, University of Florida, Gainesville, FL, United States; 2 Plant Breeding Graduate Program, University of Florida, Gainesville, FL, United States; 3 School of Plant, Environmental and Soil Sciences, Louisiana State University, Baton Rouge, LA, United States; 4 AgriLife Research, Texas A&M University, College Station, TX, United States; 5 Cereal Crops Improvement Research Unit, Edward T. Schafer Agricultural Research Center, United States Department of Agriculture - Agricultural Research Service (USDA-ARS), Fargo, ND, United States; 6 Small Grains and Potato Germplasm Research Unit, USDA-ARS, Pacific West Area, Aberdeen, ID, United States

**Keywords:** Bayesian genomic models, cross-validation strategies, disease resistance breeding, genomic selection, multi-environment prediction, oat, stem rust resistance

## Abstract

Stem rust (SR), caused by *Puccinia graminis* f. sp. *avenae*, is a major threat to oat production in the southern United States, necessitating durable resistance to sustain productivity. Genomic prediction (GP) offers a promising approach to accelerate the development of resistant cultivars by leveraging genome-wide marker data to predict breeding values. In this study, we evaluated the accuracy of genomic prediction models for SR resistance using the Southern Oat Association Panel (SOAP), a 440-line multi-institutional panel genotyped with both a 3K SNP array and genotyping-by-sequencing (GBS). Field trials were conducted across seven environments between 2022 and 2024, and disease severity (SV) and infection response (IR) were assessed. Predictive ability (PA) was estimated under three cross-validation (CV) scenarios: CV1 (untested genotypes), CV2 (incomplete field trials), and CV0 (new environments). Across traits and scenarios, Bayesian models (BayesA, BayesB, and Bayesian LASSO) consistently achieved the highest PA, with GBLUP and RRBLUP performing nearly as well, while machine-learning methods (RF and GBR) were less effective. For IR, PA was highest under CV0 (~0.55), moderate under CV2 (~0.50), and lowest under CV1 (~0.45). For SV, PA reached ~0.62 in CV0, ~0.54 in CV2, and ~0.50 in CV1. Differences between the 3K and GBS platforms were minimal, indicating that both genotyping strategies provide sufficient coverage despite contrasting marker distributions and LD patterns. These findings highlight and demonstrate the potential of GS to reduce reliance on large-scale multi-environment phenotyping, enable prediction for new genotypes and environments, and accelerate genetic gain in oat breeding programs.

## 1 Introduction

Oat (*Avena sativa* L.) serves both food and feed markets and is valued for protein, β-glucan, and bioactive phytochemicals linked to human health, while also contributing to livestock forage systems in the southern United States (US) (Pomeranz et al., 1971; Federizzi and Mundstock, 2004; Fardet, 2010; Winkler et al., 2018). Despite this versatility, the US acreage has contracted over recent decades, increasing the need for cultivars that combine milling quality with resilience to major diseases (USDA Crop Reporting Board, 1975; USDA Crop Reporting Board, 2017; McNish et al., 2020). Different diseases, including stem rust (SR), caused by *Puccinia graminis* f. sp. *avenae*, are major problems in the growth and management of winter/facultative oat in the Southern US. The pathogen damages stems, leaf sheaths, and panicles. Under conducive epidemics, SR can lead to a reduction in grain yield, forage nutritive value, and persistence. Poor quality, rust-infected forages can impact animal performance and well-being. Developing resistant cultivars is the most viable option for disease control, which shows resistance conferred by both quantitative and qualitative inheritance. Inoculum arises from barberry or long-distance spore movement, and virulent races continue to emerge, including recent predominance of TGN in parts of North America (Roelfs and Long, 1980; Wong et al., 1983; Roelfs, 1985; McCallum et al., 2000; van Niekerk et al., 2001; Peterson, 2003; USDA-ARS CDL, 2022). Race-specific resistance (qualitative) is straightforward to deploy but often short-lived, whereas partial adult-plant resistance (quantitative) tends to be more durable yet harder to select reliably (Martens, 1978; Fetch and Jin, 2007; Carson, 2008; Gnanesh et al., 2013). The improvement of quantitative resistance requires multiple cycles of selection and rigorous evaluation in well-managed screening nurseries (Rutkoski et al., 2014).

Genomic selection (GS) provides a route to accelerate genetic gain for complex resistance by predicting breeding values from genome-wide markers and shortening the selection cycle (Meuwissen et al., 2001; Gianola et al., 2006; Elshire et al., 2011; Wetterstrand, 2014). A training population with genotypes and phenotypes is used to fit whole-genome models; genomic estimated breeding values (GEBVs) are then computed for genotyped candidates, increasing gain per unit time in accordance with the breeder’s equation when cycle length is reduced (Meuwissen et al., 2001; Moose and Mumm, 2008). Prediction accuracy (PA), typically assessed by cross-validation in the training population (TP) and by a cross-environment validation, depends on marker platform and density, linkage disequilibrium (LD) with causal loci, trait architecture, heritability, TP size and structure, and the relatedness between training and validation sets (Habier et al., 2007, 2009; Daetwyler et al., 2010; Hayes and Goddard, 2010; Heffner et al., 2010; Pérez-Cabal et al., 2012; Poland et al., 2012; Resende et al., 2012; de los Campos et al., 2013; Lin et al., 2014).

Because marker resources differ in density, reproducibility, and cost, both genotyping-by-sequencing (GBS) and targeted array SNPs can be used. GBS offers broad genome coverage and many informative variants, while a curated mid-density panel provides standardized, high-quality markers with rapid turnaround. Theory and empirical results indicate that high relatedness between training and target sets and adequate linkage disequilibrium can allow mid-density panels to perform competitively, whereas sparser LD or weaker relatedness may require higher density to sustain accuracy (Daetwyler et al., 2010; Heffner et al., 2010; de los Campos et al., 2013; Lin et al., 2014).

Multiple statistical learning frameworks are relevant for predicting quantitative disease resistance. Linear mixed models, such as ridge-regression best linear unbiased prediction (RRBLUP) and genomic best linear unbiased prediction (GBLUP), assume many small additive effects and leverage either marker shrinkage or a genomic relationship matrix to share information among related individuals (Henderson, 1975; Whittaker et al., 2000; Habier et al., 2007).Bayesian regressions such as BayesA, BayesB, and Bayesian LASSO (BL) permit heterogeneous shrinkage or sparse solutions, which can be advantageous when a subset of loci contributes more strongly to variation (Meuwissen et al., 2001; Park and Casella, 2008). Nonlinear and machine-learning methods, including Random Forest (RF) and Gradient Boosting (GBR), can capture departures from additivity and interactions among markers (Breiman, 2001). Prior studies in quantitative disease resistance often report RRBLUP performing on par with Bayesian LASSO and BayesB, with occasional gains from kernel or tree-based methods depending on trait architecture and data structure (Faé et al., 2009; Lorenz et al., 2012; Ornella et al., 2012; Rutkoski et al., 2012, 2014; Arruda et al., 2015; Sallam et al., 2015).

Within this context, genomic prediction is applied to the Southern Oat Association Panel (SOAP), a 440-entry public-program panel adapted to the southern US with the following objectives: (i) compare GBLUP, RRBLUP, BayesA, BayesB, BL, RF, and GBR models for genomic prediction across multiple environments using GBS and a 3K SNP array; (ii) cross-validate the prediction accuracy (PA) of models in different scenarios (CV2, CV1, and CV0).

## 2 Materials and methods

### 2.1 Plant materials and field experiment

The study was conducted using 440 elite oat genotypes from the SOAP, a collaborative resource developed within the SunGrains^®^ breeding network. The panel represents advanced selections and breeding lines contributed by five public programs in the southern US (University of Florida, Louisiana State University, North Carolina State University, Clemson University, and Texas A&M University). Most entries are winter or facultative types, supplemented with a small set of historical accessions, thereby capturing the genetic diversity relevant to oat improvement in the region.

Field experiments were carried out for three consecutive years (2022–2024) in environments with a history of SR epidemics. In 2022, 300 genotypes were grown at Citra, Florida (22_CF) using a randomized complete block design (RCBD) with three replications. In 2023, the evaluation expanded to four environments: Citra, FL (23_CF), Castroville, TX (23_CT), Baton Rouge, LA (23_BL), and Winnsboro, LA (23_WL). Each location included a full panel of 440 lines, with three replications at CF and CT and two replications at BL and WL. In 2024, trials were repeated at CF, BL, and CT with three replications per site. To enhance and standardize inoculum levels, susceptible cultivars (Brooks and RAM 99016) were used as border rows. Plots consisted of single 1.2 m rows with 36 cm spacing, sown using a head-row planter with approximately 3 g of seed per row to ensure consistent stand establishment.

### 2.2 Genotyping

Genotyping for the SOAP panel used two platforms: GBS and the USDA SoyWheOatBar 3K oat SNP array (3K), performed at the USDA–ARS Genotyping Laboratory in Fargo, ND. Seeds of each accession were germinated in pots filled with a standard substrate, and young leaves were harvested at the seedling stage. Collected tissue was placed into 96-well plates containing silica sand to facilitate rapid drying and preservation before DNA extraction. Genomic DNA was isolated following a detergent-based protocol that included lysis with 10% sodium dodecyl sulfate at 65 °C, precipitation of proteins using 6 M ammonium acetate, DNA precipitation with chilled 70% isopropanol, and a final wash with 70% ethanol.

Library preparation followed a reduced-representation method in which *PstI* and *MspI* restriction enzymes were used, and individual samples were distinguished by unique barcode adapters. Sequencing was performed on the Illumina HiSeq 2000 platform, producing 150 bp paired-end reads. Adapter sequences and low-quality bases were trimmed in bbduk (https://sourceforge.net/projects/bbmap/) using the parameters ktrim=r, k=23, mink=11, and hdist=1. Reads that passed quality filtering were aligned to the *Avena sativa* OT3098 v2 PepsiCo reference genome (https://wheat.pw.usda.gov/jb?data=/ggds/oat-ot3098v2-pepsico) using Bowtie2 (Poland et al., 2012). Alignment files were sorted and indexed with SAMtools (Danecek et al., 2021), and SNP discovery was performed using SAMtools and BCFtools v1.14 (Li, 2011). Variants were retained only if they met thresholds of mapping quality ≥ 30 and read depth ≥ 4.

From the 51,534 SNPs initially detected, multiallelic sites were removed, and only biallelic loci were retained. Additional filtering excluded variants with a minor allele frequency (MAF)< 5%, heterozygosity > 10%, or lacking an assigned chromosomal position. Markers missing in more than 20% of accessions were also removed. The remaining missing data were imputed using the LD-kNNi algorithm in TASSEL v5.0 (Bradbury et al., 2007). After all filtering steps, 12,914 SNP markers were retained for subsequent analyses from GBS and 2120 SNPs from the 3K-array.

### 2.3 Phenotyping and statistical analysis

The SR evaluations were carried out from the milk to the early dough stages of plant development. Two complementary traits were assessed: disease severity (SV) and infection response (IR). Severity was measured as the proportion of stem area (0–100%) covered by uredinia, using the modified Cobb scale (Peterson et al., 1948). Infection response was scored according to the system of Roelfs and Long (1980), which classifies host reactions based on pustule size and associated chlorosis or necrosis. Reaction categories included immune (0), resistant (R), moderately resistant (MR), moderately susceptible (MS), and susceptible (S). When plots exhibited mixed reactions, composite scores were assigned based on the dominant response. For statistical purposes, categorical IR scores were converted to numeric values: 0.0 (immune), 0.2 (R), 0.4 (MR), 0.8 (MS), and 1.0 (S), with intermediate values averaged in cases of mixed reactions (Stubbs et al., 1986).

In addition to rust ratings, heading date (HD) was recorded as the number of days from planting to 50% heading, expressed in Julian calendar days (Zadoks et al., 1974)In environments other than Citra, FL, relative maturity (RM) was scored on a five-point scale: 1 (booting), 2 (heading), 3 (milk stage), 4 (soft dough), and 5 (hard dough). HD or RM were incorporated as a continuous covariate in subsequent analyses to account for maturity-related effects.

Descriptive statistics for all traits were generated separately for each environment (location-year combination) using the dplyr package in R. Analysis of variance (ANOVA) was performed to test the contribution of genotype and replication within each environment. Since replication effects were significant in some environments, least squares (LS) means were estimated with the emmeans package and used as the adjusted phenotypic values. LS means were computed both for individual environments and for the combined dataset across environments.

For single-environment analyses, the following linear model (Equation 1) was fitted:

(1) $$y_{ij}=\mu +g_{i}+r_{j}+\beta .HD_{ij}+\epsilon_{ij}$$

where *y_ij_* denotes observed trait value for the genotype *i* in the replication *j*, μ is the overall mean, *g_i_* is the fixed effect of genotype, *r_j_* is the fixed effect of replication, *HD_ij_* is the covariate for the heading date or relative maturity, *β* is the regression coefficient for the covariate, and $\epsilon_{ij}\sim N\left(0,\sigma_{R}^{2}\right)$ is the residual error. This model was implemented using the lm() function in R, and LS means for each genotype were extracted using the emmeans package.

To estimate the combined LS means for all traits, a linear mixed-effects model was fitted using the lmer() function from the lme4 package in R. Each location-year combination is considered as one environment. The model was specified as Equation 2:

(2) $$y_{ijk=}\mu +G_{i\;+}E_{j}+R_{jk}+\beta .HD_{ijk}+\epsilon_{ijkl}$$

where *y_ijk_* denotes observed trait value for the genotype *i* in the replication *j* in the environment *k*, μ is the overall mean*, g_i_* is the fixed effect of *i*^th^ genotype, *r_jk_* is the effect of replication nested within the environment, *HD_ijk_* is the covariate for the heading date or relative maturity, *β* is the regression coefficient for the covariate, and $\epsilon_{ijk}\sim N\left(0,\sigma_{R}^{2}\right)$ is the residual error.

Broad-sense heritability *(H²)* was estimated for each trait both within individual environments (i.e., each unique combination of year and location) and across all environments combined using linear mixed models. For each environment, broad-sense heritability was estimated using the following equations (Equations 3, 4)

(3) $$H^{2}=\frac{{\text{σ}}_{G}^{2}}{{\text{σ}}_{G}^{2}+{\text{σ}}_{\frac{e}{r}}^{2}}$$

(4) $$H^{2}=\frac{{\text{σ}}_{G}^{2}}{{\text{σ}}_{G}^{2}+{\text{σ}}_{\frac{GE}{e}}^{2}+{\text{σ}}_{\frac{e}{er}}^{2}}$$

Where ${\text{σ}}_{G}^{2}$ represents the genotypic variance, ${\text{σ}}_{GE}^{2}$ denotes the genotype-by-environment interaction variance, and ${\text{σ}}_{e}^{2}$ corresponds to the error variance. The terms r and e refer to the number of replications and environments, respectively. Variance components were estimated using the lme4 package.

### 2.4 Linkage disequilibrium

The LD was evaluated in TASSEL v5.0 (Bradbury et al., 2007) by calculating pairwise r² values between SNP markers within a sliding window of 50 markers. The decay of LD with physical distance (bp) was assessed in R by fitting a nonlinear regression model following Hill and Weir (1988). Pairwise r² values were plotted against marker distance, and a LOESS curve was fitted to visualize the decay. The extent of LD was defined as the physical distance at which the LOESS regression of mean r² dropped below 0.2, a commonly used threshold in related studies (Zhu et al., 2008; Bazzer et al., 2025)

### 2.5 Genomic prediction models and validation

Predictive ability (PA) for SR severity (SV) and infection response (IR) was assessed using seven genomic prediction models: Genomic Best Linear Unbiased Prediction (GBLUP), Ridge Regression Best Linear Unbiased Prediction (RR-BLUP) (Meuwissen et al., 2001), BayesA (Meuwissen et al., 2001), BayesB (Meuwissen et al., 2001), Bayesian Lasso (BL) (Park and Casella, 2008), Random Forest (RF) (Chen and Ishwaran, 2012), and Gradient Boosting Regression (GBR) (Ridgeway, 2007). Models were implemented using the R packages BGLR (Pérez and de los Campos, 2014), randomForest (Liaw and Wiener, 2002), and gbm (Ridgeway, 2007).

#### 2.5.1 GBLUP

The GBLUP model was fitted using BGLR (Equation 5):

(5) y=μ1+Zɡ+e

where y is an n×1 vector containing the ls means for each trait; μ denotes the overall mean; Z is the incidence matrix linking observation to genotypic effects; The vector g represents normally distributed marker predictor effects, with $g\sim N\left(0,G\sigma_{ɡ}^{2}\right)$, where *G* is the genomic relationship matrix and $\sigma_{ɡ}^{2}$ denotes the additive genetic variance; The residuals e are assumed to be normally distributed with ***e*** *~ N(0*, $\sigma_{e}^{2}$), where $\sigma_{e}^{2}$ is the residual variance

#### 2.5.2 RR-BLUP

The RR-BLUP model was also implemented in BGLR (Equation 6):

(6) y=μ1+Xβ+e

where y, *μ*, are as in Equation 5, X is the marker genotype matrix (*n* individuals × *p* SNP markers), β is a vector of marker effects assumed to follow a normal distribution with common variance $\beta_{j}\sim N\left(0,\sigma_{\beta }^{2}\right)$, *e* is a vector of residuals assumed to be normally distributed ***e*** *~ N(0,I* $\;\sigma_{e}^{2}$).

#### 2.5.3 BayesA

BayesA (Meuwissen et al., 2001) was also implemented in BGLR (Equation 7). Its formulation is:

(7) y=μ1+Xβ+e

where y, *μ*, and e are as in Equations 5, 6; X is the genotype matrix (*n* individuals × *p* markers) and β is the corresponding vector of marker effects. In BayesA, each marker effect $\beta_{j}$ is assumed to follow a normal distribution with a marker-specific variance:


$$\beta_{j}\sim N\left(0,\sigma_{\beta_{j}}^{2}\right),\sigma_{\beta_{j}}^{2}\sim Scaled-inverse-\chi 2\left(V,S\right)$$

where, $\sigma_{\beta_{j}}^{2}$ is the variance of the *j*^th^ marker effect, drawn from a scaled-inverse chi-squared distribution with ν degrees of freedom and scale parameter S. This hierarchical prior induces a scaled t-distribution on the marginal distribution of marker effects, allowing each marker to have its own level of shrinkage. The GEBVs are obtained as the linear combination of estimated marker effects.

#### 2.5.4 BayesB

BayesB (Meuwissen et al., 2001) was also implemented in BGLR (Equation 8). Its formulation is:

(8) y=μ1+Xβ+e

with a mixture prior on marker effects:


$$\beta_{j}\sim \pi \delta_{0}+\left(1-\pi \right)\;N\left(0,\sigma_{\beta_{j}}^{2}\right)$$

where $\delta_{0}$ is a point mass at zero, and π is the prior probability that a marker has no effect. Nonzero effects have variances $\sigma_{\beta_{j}}^{2}$ drawn from a scaled inverse chi-squared distribution. This specification enforces sparsity, setting many marker effects to zero while allowing a subset of influential markers to explain variation.

#### 2.5.5 Bayesian lasso

The Bayesian Lasso (Park and Casella, 2008) was also fitted in BGLR (Equation 9). The model is:

(9) $$\beta_{j}\sim N\left(0,\tau_{j}^{2}\right)\;\tau_{j}^{2}\sim Exponential\;\left(\frac{\lambda^{2}}{2}\right)$$

with marker effects assigned a double-exponential (Laplace) prior: $\beta_{j}\sim DE\left(\lambda \right)$This can be represented hierarchically as: y=μ1+Xβ+ewhere λ controls the shrinkage intensity. The BL approach shrinks small effects strongly toward zero while permitting larger effects to remain, yielding a continuous sparsity-inducing alternative to the discrete BayesB prior.

#### 2.5.6 Random forest

RF was implemented in R using the randomForest package (Liaw and Wiener, 2002). Predictions are obtained as the ensemble mean across N decision trees (Equation 10):

(10) $${\hat{y}}_{i}=\frac{1}{N}\sum_{n=1}^{N}h_{n}\left(x_{i}\right)$$

where ${\hat{y}}_{i}$ is the predicted value for individual *i* with input features $x_{i}$ (i.e., marker data), N is the total number of trees in the forest, and $h_{n}\left(x_{i}\right)$ represents the prediction from the *n*^th^ tree. Each tree is trained on a bootstrap sample of the training data, and at each split, a random subset of markers is evaluated. The final prediction is the average across all trees, improving robustness and capturing nonlinear interactions.

#### 2.5.7 Gradient boosting regression

GBR was applied in R using the gbm package (Ridgeway, 2007). Predictions are constructed sequentially across *M* trees (Equation 11):

(11) $${\hat{y}}_{i}=\sum_{m=1}^{M}\alpha h_{m}\left(x_{i}\right)$$

where ${\hat{y}}_{i}$ is the predicted outcome for individual *i* with marker data $x_{i}$, M is the total number of boosting iterations (trees), α is the learning rate that controls the contribution of each tree, and $h_{m}\left(x_{i}\right)\;$ represents the prediction from the *m*^th^ tree. Each successive tree is trained on the residuals from the model built up to that point, progressively reducing prediction error. Bootstrap sampling and random feature subsets were used at each stage, and partitions were chosen to minimize mean squared error (MSE). In this study, M = 500 iterations were used, with α providing regularization.

#### 2.5.8 Cross-validation strategies

Model performance was evaluated using three cross-validation (CV) schemes as described by Jarquín et al. (2014).

- CV1 (genotype-based): Fivefold CV was applied with all observations of a given genotype assigned to a single fold. This design mimics predicting new, untested genotypes.
- CV2 (plot-based): Records were divided at the plot level, allowing different environments of a genotype to be split across folds. This simulates incomplete multi-environmental trials.
- CV0 (environment-based): A leave-one-environment-out approach was used, training on six environments and predicting in the seventh. This tests the ability to forecast performance in unobserved environments.

For CV1 and CV2, 80% of the data was used for training and 20% for testing in each of five folds. Predictive ability (PA) was quantified as the Pearson correlation between LS means and genomic predictions. For CV0, 6 environments were used to predict the seventh environment.

## 3 Results

### 3.1 Heritability and phenotypic analysis

Results from individual environments (**Table 1**) highlight clear differences in the genetic signal for SR resistance across locations and years. At CF, moderate heritability was observed in 2022 (*H²* = 0.39 for IR; 0.52 for SV), while estimates increased in 2023 (*H²* = 0.77 for IR; 0.82 for SV). In BL, heritability values were consistently high, reaching 0.83–0.89 for IR and 0.87–0.89 for SV across 2023 and 2024. Similarly, CT exhibited strong genetic control, with heritability estimates ranging from 0.82–0.90 for IR and 0.92–0.93 for SV in 2023 and 2024. By contrast, WL in 2023 showed weak genetic differentiation, with IR being non-significant (*p* = 0.0876) and heritability estimates of 0.19 for IR and 0.27 for SV, likely to reflect low or uneven disease pressure. In the combined analysis across seven environments, *H²* was 0.87 for SV and 0.81 for IR (**Supplementary Table 1**), indicating substantial genetic control of phenotypic variance.

**Table 1:** Descriptive statistics of the southern oat association panel (SOAP) in response to stem rust at the adult stages for seven location years.

Location	Year	Trait	Min	Max	Mean	SD	*H^2^*	*p*-value^a^
CF	2022	IR	0	1	0.98	0.10	0.39	<0.0001
	2022	SV	0	90	84.6	13.6	0.52	<0.0001
	2023	IR	0	1	0.93	0.16	0.77	<0.0001
	2023	SV	10	90	71.7	20.9	0.82	<0.0001
BL	2023	IR	0	1	0.95	0.11	0.83	<0.0001
	2023	SV	10	90	75.9	19.0	0.87	<0.0001
	2024	IR	0	1	0.89	0.20	0.88	<0.0001
	2024	SV	5	80	54.4	20.9	0.89	<0.0001
CT	2023	IR	0	1	0.98	0.08	0.9	<0.0001
	2023	SV	10	90	84.7	16.3	0.93	<0.0001
	2024	IR	0	1	0.95	0.14	0.82	<0.0001
	2024	SV	0	90	72.1	20.3	0.92	<0.0001
WL	2023	IR	0	1	0.91	0.19	0.19	0.0876
	2023	SV	0	90	67.2	21.5	0.27	<0.0001

IR, infection response; and SV, disease severity.

a *p*-value obtained from the analysis of variance (ANOVA) table. If *p* < 0.05, the oat lines are significantly different for the trait in that environment. CF, BL, CT, and WL indicate Citra, Florida; Baton Rouge, Louisiana; Castroville, Texas; and Winnsboro, Louisiana, respectively. Each location-year combination is one environment.

Pairwise Pearson correlation coefficients were calculated among location–year combinations using genotype least-squares means to evaluate the stability of stem rust responses across environments (**Supplementary Figure 1**). For both infection response (IR) and disease severity (SV), most environments displayed moderate to strong positive correlations, particularly between CT and BL in 2023 and 2024 (IR: r = 0.49–0.71; SV: r = 0.54–0.79), suggesting consistent genotype performance under similar disease pressure conditions. In contrast, the 23_WL environment showed weak to negligible correlations with all other trials for both traits (IR: r = −0.07 to 0.02; SV: r = −0.04 to 0.01). These near-zero correlations align with its lower heritability estimates and irregular disease expression. Collectively, these results indicate the presence of genotype × environment interaction, while also demonstrating strong agreement among environments with stable and reliable disease pressure.

### 3.2 Linkage disequilibrium and marker coverage

**Figure 1:**
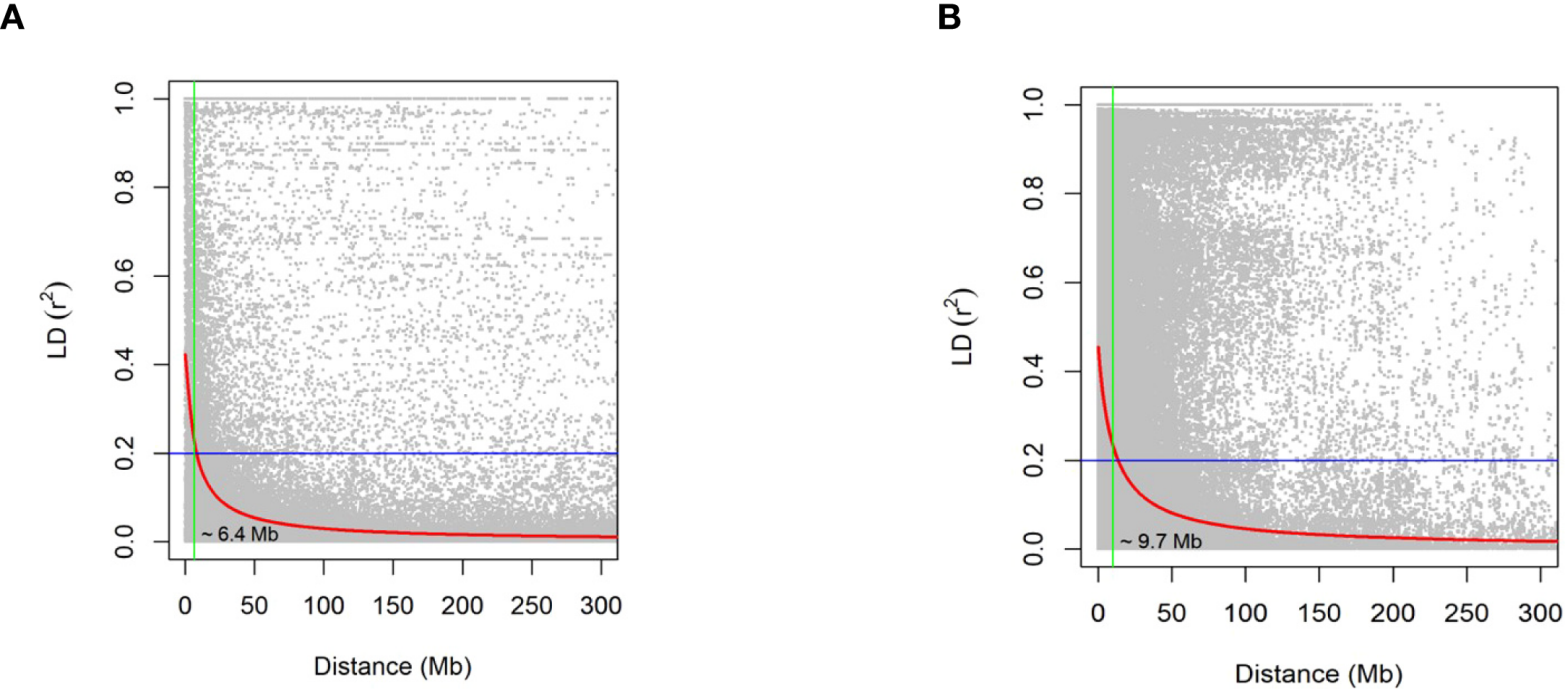
Linkage disequilibrium (LD) decay plot for the southern oat association mapping panel (SOAP). Pairwise correlation (*r*^2^) between markers on all 21 oat nuclear chromosomes was calculated as a metric for LD. The locally estimated scatterplot smoothing (LOESS) curves summarize LD on the whole genome. The blue horizontal line indicates the *r*^2^ threshold of 0.2. Figure **(a)** shows 3K data with 2134 SNPs, and Figure **(b)** shows GBS data with 12914 SNPs.

Across the two genotyping platforms, clear differences were observed in the distribution of markers among the three oat subgenomes. In contrast, the 3K array yielded 2,134 SNPs, with 751 markers in the A subgenome, 736 in the C subgenome, and 647 in the D subgenome, representing a more balanced coverage across the three subgenomes (**Supplementary Figure 2**). In the GBS dataset, a total of 12,914 high-quality SNPs were retained, of which 2,949 mapped to the A subgenome, 6,416 to the C subgenome, and 3,549 to the D subgenome (**Supplementary Figure 3**). This distribution indicates an enrichment of polymorphic sites within the C subgenome, which accounted for nearly half of all markers. These differences highlight platform-specific variation in marker density, with GBS offering higher overall marker numbers but a skew toward the C subgenome, while the 3K array provided more even representation across the A, C, and D subgenomes. LD analysis revealed contrasting decay patterns between the two marker platforms (**Figure 1**). Using the r² = 0.2 threshold as a benchmark, LD in the 3K array extended to approximately 6.4 Mb, whereas the GBS dataset exhibited a slower decay, persisting until ~9.7 Mb.

### 3.3 Predictive ability and cross-validation

#### 3.3.1 IR

**Figure 2:**
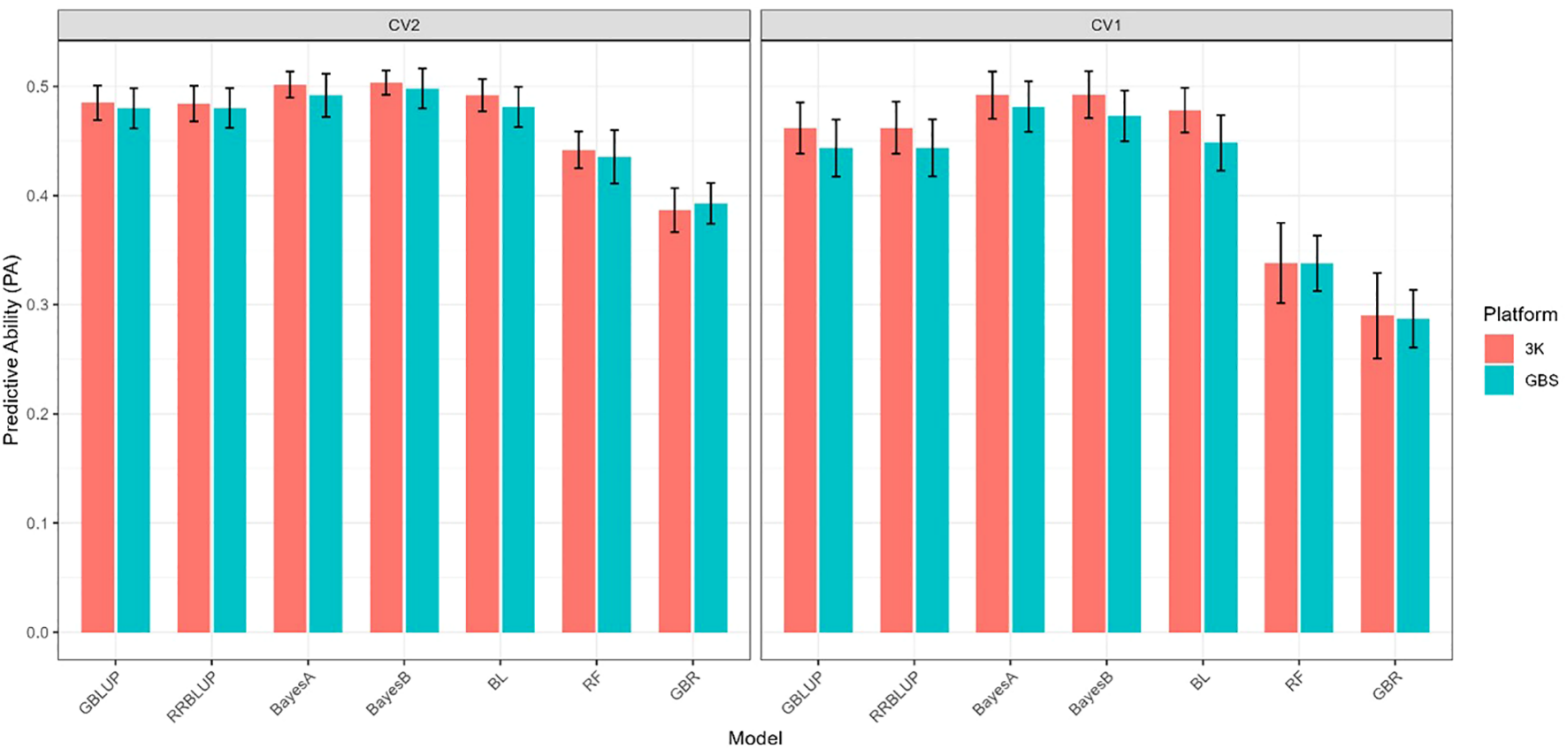
Predictive ability of seven genomic prediction models (GBLUP, RRBLUP, BayesA, BayesB, BL, RF, and GBR) for trait IR using two marker platforms (3K and GBS). Results are shown under two cross-validation schemes (CV2 and CV1).

For IR (**Figure 2**), the PA under CV2, which simulates incomplete field trials, was moderate across parametric models, with values clustering between 0.48 and 0.50. The Bayesian models (BayesA and BayesB) performed slightly better (PA = 0.50–0.51 with 3K; 0.49–0.50 with GBS), followed closely by BL, GBLUP, and RRBLUP. RF achieved slightly lower PA (~0.44), while GBR was consistently the least accurate (~0.39). Across all models, differences between the 3K and GBS panels were minor, though 3K often provided a small advantage.

Under CV1 (**Figure 2**), where entire genotypes were excluded from training, PA values decreased across models, reflecting the greater difficulty of predicting untested lines. Parametric models achieved PA values of 0.44–0.46 (GBLUP/RRBLUP) and 0.48–0.49 (BayesA/BayesB), with BL performing similarly. RF and GBR showed the largest declines, with PA dropping to ~0.33 and ~0.29, respectively. Consistent with CV2, the 3K panel generally outperformed GBS by a narrow margin.

**Figure 3:**
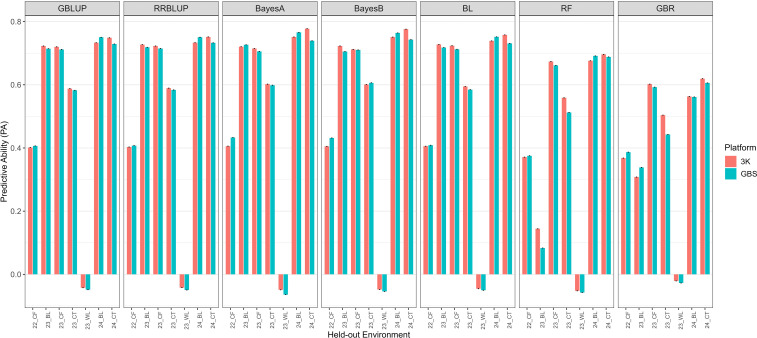
Predictive ability of seven genomic prediction models (GBLUP, RRBLUP, BayesA, BayesB, BL, RF, and GBR) for trait IR under CV0 (leave-one-environment-out) cross-validation. Each bar represents the predictive ability for a held-out environment (Loc) across years and locations, comparing two marker platforms (3K and GBS). Environment codes combine year and location: 22CF = Citra, FL in 2022; 23CF = Citra, FL in 2023; 23CT = Castroville, TX in 2023; 23BL = Baton Rouge, LA in 2023; 23WL = Winnsboro, LA in 2023; 24BL = Baton Rouge, LA in 2024; 24CT = Castroville, TX in 2024. Error bars are not shown because each environment in CV0 represents a single prediction iteration (leave-one-environment-out), providing only one accuracy estimate per environment.

In the CV0 scheme (**Figure 3**), which evaluates prediction across environments, the PA was slightly higher overall but more variable depending on the held-out environment. Mean PA values across parametric models were 0.55–0.56 for both platforms, with BayesA, BayesB, and BL achieving the highest performance. GBLUP and RRBLUP were only marginally lower. Machine-learning models again showed weaker and less stable results, with RF averaging ~0.42–0.44 and GBR ~0.41–0.42. Notably, in WL 2023, PA occasionally approached zero or became negative. Although this environment exhibited low heritability and uneven disease pressure, pairwise Pearson correlations among environments (**Supplementary Figure 1**) revealed near-zero concordance between WL 2023 and all other trials, indicating substantial genotype × environment interaction and rank changes. Because across-environment prediction depends on shared genetic covariance between training and testing environments, this weak concordance likely contributed to the unstable and occasionally negative PA observed under CV0. Importantly, CV0 results were nearly identical between platforms, suggesting that when predicting new environments, platform choice had minimal impact in this dataset.

For IR, predictive ability was highest under CV0, followed by CV2, and lowest under CV1. Among models, Bayesian approaches (BayesA, BayesB, and BL) consistently delivered the strongest performance, with GBLUP and RRBLUP performing comparably well, whereas machine-learning methods showed reduced accuracy, particularly under CV1.

#### 3.3.2 SV

**Figure 4:**
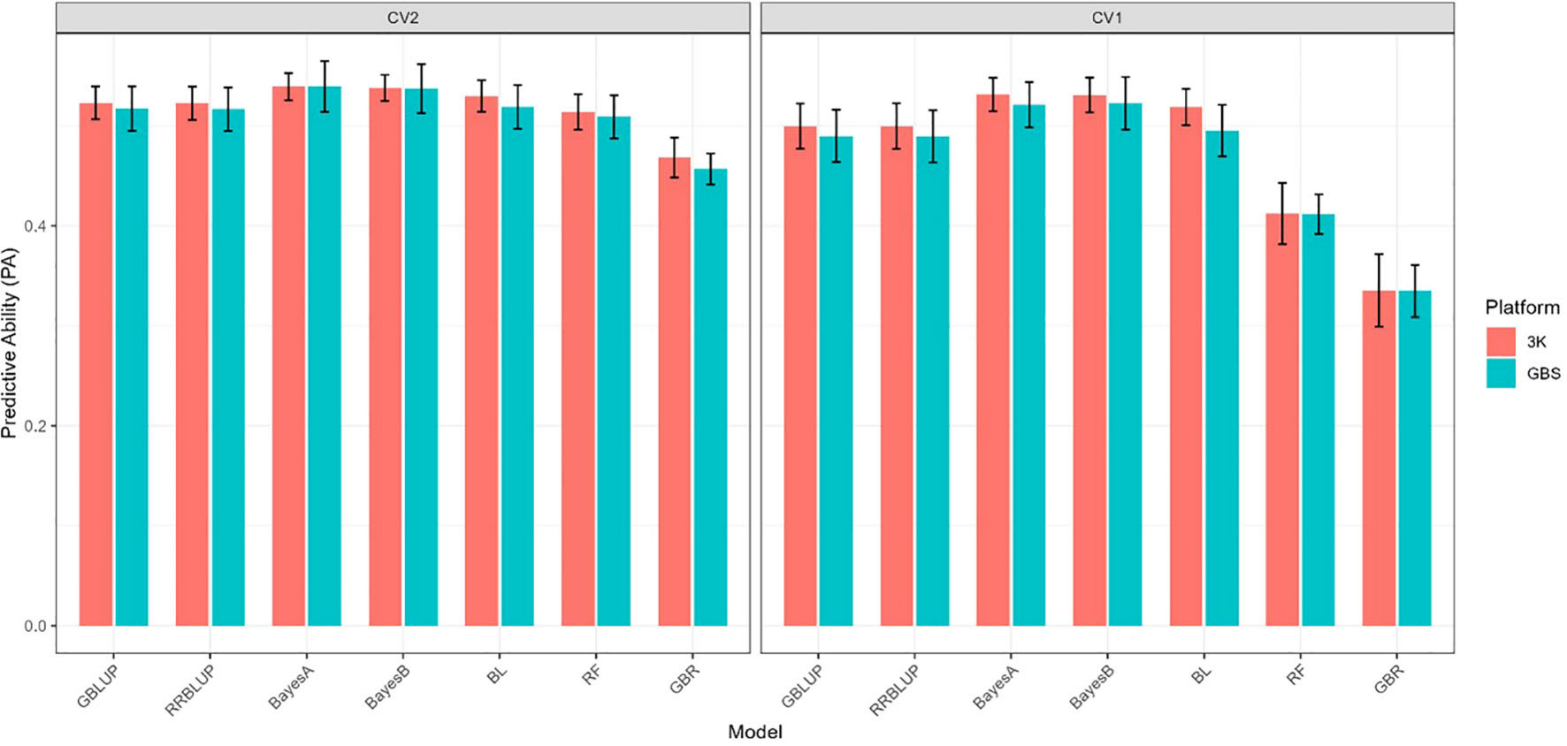
Predictive ability of seven genomic prediction models (GBLUP, RRBLUP, BayesA, BayesB, BL, RF, and GBR) for SV using two marker platforms (3K and GBS). Results are shown under two cross-validation schemes (CV2 and CV1).

For SV (**Figure 4**), PA under CV2 was consistently higher than for IR, with parametric models ranging from 0.52 to 0.54. Bayesian models (BayesA and BayesB) again performed slightly better (PA = 0.54 with 3K and 0.53–0.54 with GBS), while BL, GBLUP, and RRBLUP followed closely at 0.52–0.53. Machine-learning models were less accurate, with RF averaging ~0.51 and GBR ~0.46–0.47. Across models, the 3K and GBS platforms produced nearly identical results, with only marginal differences favoring 3K in some cases.

Under CV1 (**Figure 4**), where prediction is made for entirely untested lines, PA values decreased relative to CV2 but remained moderate. GBLUP and RRBLUP averaged ~0.50 with 3K and ~0.49 with GBS, while Bayesian models achieved slightly higher values (0.53 with 3K; 0.52 with GBS). BL also performed well, but machine-learning models showed the largest declines, with RF averaging ~0.41–0.42 and GBR ~0.33–0.34. Similar to IR, the 3K panel provided a small but consistent edge over GBS.

**Figure 5:**
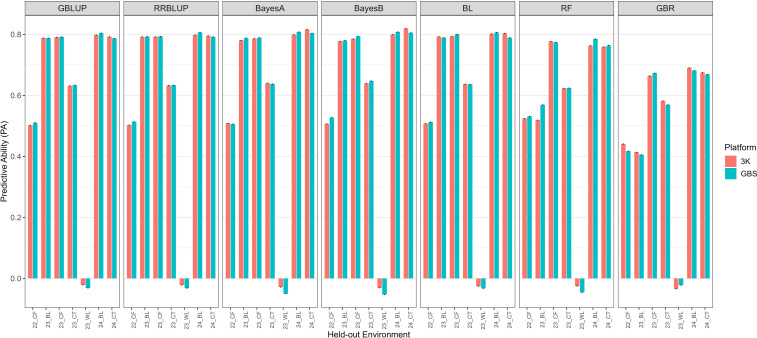
Predictive ability of seven genomic prediction models (GBLUP, RRBLUP, BayesA, BayesB, BL, RF, and GBR) for trait SV under CV0 (leave-one-environment-out) cross-validation. Each bar represents the predictive ability for a held-out environment (Loc) across years and locations, comparing two marker platforms (3K and GBS). Environment codes combine year and location: 22CF = Citra, FL in 2022; 23CF = Citra, FL in 2023; 23CT = Castroville, TX in 2023; 23BL = Baton Rouge, LA in 2023; 23WL = Winnsboro, LA in 2023; 24BL = Baton Rouge, LA in 2024; 24CT = Castroville, TX in 2024. Error bars are not shown because each environment in CV0 represents a single prediction iteration (leave-one-environment-out), providing only one accuracy estimate per environment.

In the CV0 scheme (**Figure 5**), which assesses prediction across environments, PA values were generally highest, reflecting the stronger genetic signal for SV. Mean PA across Bayesian and parametric models reached 0.61–0.62 on both platforms, with BL slightly outperforming BayesA and BayesB in some environments. GBLUP and RRBLUP were only marginally lower. RF showed moderate PA (~0.56–0.57), while GBR trailed at ~0.48–0.49. However, predictions for WL 2023 were unstable and occasionally negative. As shown in **Supplementary Figure 1**, WL 2023 exhibited near-zero correlations with the remaining environments, supporting the presence of strong genotype × environment interaction and substantial rank reshuffling. The limited genetic concordance between this environment and the training set likely explains the reduced predictive performance under the leave-one-environment-out scheme. Similar to IR, platform differences under CV0 were negligible.

For SV, predictive ability is ranked CV0 > CV2 > CV1. Bayesian models (BayesA, BayesB, and BL) again achieved the strongest and most consistent results, closely followed by GBLUP and RRBLUP, whereas machine-learning approaches were less effective, especially under CV1.

## 4 Discussion

### 4.1 Heritability and phenotypic analysis

In this study, substantial variation in SV and IR was detected across multiple environments spanning three years, and heritability estimates reflected clear differences in the strength of the genetic signal among locations. Moderate values at CF in 2022 increased markedly in 2023, while BL and CT consistently exhibited high heritability across years, underscoring the reliability of these sites for discriminating against genetic variation. In contrast, WL in 2023 showed weak or non-significant genetic effects, consistent with limited or uneven disease pressure in that environment (**Table 1**). Importantly, when data were combined across all environments, heritability remained high (**Supplementary Table 1**), demonstrating that aggregation across diverse trials improved the ability to partition genetic variance rather than diluting it through genotype-by-environment interactions. These results are consistent with previous reports in global wheat germplasm, where *H²* values for rust resistance were similarly moderate to high (Gao et al., 2017; Shewabez et al., 2022). Together, the findings highlight that the SR resistance in the SOAP panel is under strong genetic control and that reliable multi-environmental data provide a robust foundation for genomic prediction.

### 4.2 Linkage disequilibrium and marker coverage

Our comparison of the 3K SNP array and GBS revealed clear differences in marker distribution and LD structure; however, these differences did not translate into meaningful differences in genomic prediction accuracy. The 3K array provided a more balanced representation across the A, C, and D subgenomes, whereas GBS delivered substantially higher overall marker density but was enriched in the C subgenome. Such skewed marker distributions have been reported in other cereals, where genotyping-by-sequencing generates uneven coverage due to differences in genome size, repeat content, and polymorphism rates among subgenomes (Romay et al., 2013; He et al., 2014). In oat, the C genome has been reported to be larger and more polymorphic than the A or D genomes (Yan et al., 2016; Bekele et al., 2018), which likely contributes to the enrichment of C-genome markers observed in the GBS dataset. Similar observations were reported by Argenta et al. (2025), who used DArT markers and highlighted aspects related to C genome coverage.

Despite this imbalance, predictive ability under all cross-validation schemes was nearly identical between platforms, suggesting that genomic selection in this elite panel is driven primarily by genome-wide relationship capture and linkage disequilibrium between markers and causal loci rather than equal marker representation across subgenomes. In structured breeding populations with moderate LD, genomic prediction accuracy often depends more on capturing realized relatedness than on uniform genome coverage (Meuwissen et al., 2001; Hayes and Goddard, 2010). While it remains possible that important stem rust resistance loci are enriched in the C subgenome, the comparable performance of both platforms indicates that subgenome bias did not penalize prediction accuracy in this dataset and may be more consequential for locus discovery than for genomic selection.

LD decay patterns also differed between platforms, with LD extending to ~9.7 Mb in GBS and ~6.4 Mb in the 3K array. Although higher marker density is typically expected to reveal shorter LD decay due to finer resolution of haplotype blocks (Hao et al., 2011; Juliana et al., 2022), LD estimates derived from GBS data can be influenced by higher levels of missingness, imputation procedures, minor allele frequency filtering, and uneven marker spacing. Imputation-based approaches have been shown to inflate r² under certain conditions, particularly when markers are clustered within genomic regions (Swarts et al., 2014; Browning and Browning, 2016). Therefore, the longer LD observed in the GBS dataset likely reflects a combination of biological LD and platform-specific properties rather than a true extension of haplotype blocks. Importantly, because both platforms produced similar predictive ability, our results indicate that genomic selection in this population is robust to these platform-specific differences in marker distribution and LD estimation.

### 4.3 Genomic prediction

Several studies have demonstrated that implementing GS in breeding programs can shorten the breeding cycle, enhance selection accuracy, accelerate genetic gain per unit time and cost, and reduce the need for extensive multi-environment phenotyping of breeding lines (Heffner et al., 2010; Jarquín et al., 2014; Heslot et al., 2015). Given the rapid expansion of oat genomic research in recent years (Avni et al., 2026), the value of genomic prediction have been demonstrated in oat for agronomic traits including crown rust severity (Oliveira et al., 2025) as well as biomass yield (Adewale et al., 2026). In this context, GS enables the improvement of germplasm by predicting line performance under three commonly applied scenarios, CV2, CV1, and CV0, in plant breeding (Burgueño et al., 2012). The application of GS to SR in oats builds on the moderate to high heritability observed across environments in this panel, indicating that substantial additive genetic variance is available for prediction. Consistent with theoretical expectations (Meuwissen et al., 2001) and previous empirical studies in cereals (Rutkoski et al., 2014; Poland and Rutkoski, 2016; Azizinia et al., 2020), linear parametric models captured the majority of this signal. In particular, Bayesian approaches (BayesA, BayesB, and Bayesian LASSO) consistently delivered the highest and most stable predictive abilities across CV scenarios for both IR and SV, with GBLUP and RRBLUP performing nearly as well. These outcomes suggest that SR resistance in this population is largely additive in nature, a pattern also reported for rust resistance in wheat (Crossa et al., 2010; Juliana et al., 2017), where genome-wide linear models reliably capture the genetic architecture of quantitative resistance.

Machine learning algorithms such as RF and GBR were less effective in our study, especially under CV1, where the prediction of entirely untested genotypes proved challenging. Similar underperformance of tree-based methods has been observed in other crops (Howard et al., 2014; Perez et al., 2022), often attributed to their sensitivity to high-dimensional but largely additive data structures. The weaker performance of these models in our study suggests that non-additive interactions play a limited role in SR resistance in this panel of germplasm, aligning with the relatively stable genetic signal detected across environments in the genome-wide association study (GWAS) in this panel using the GBS SNPs (Acharya et al., 2026). The reduced performance of machine-learning models under CV1 likely reflects their sensitivity to training-set structure and potential overfitting to relationships among observed genotypes. In the CV1 scheme, predictions are made for entirely untested lines that may share limited relatedness with the training population. Parametric models such as GBLUP and Bayesian approaches primarily capture additive genetic relationships and are therefore more robust when extrapolating to unrelated individuals. In contrast, tree-based machine-learning methods such as RF and GBR can model complex and non-linear interactions but may rely heavily on patterns specific to the training set, resulting in reduced predictive ability when applied to novel genotypes. This behavior has been widely reported in genomic prediction studies and underscores the importance of model choice depending on the intended breeding application.

The differences among cross-validation schemes are also informative for breeding applications. CV1 predictably yielded the lowest accuracy, underscoring the challenge of extrapolating to entirely novel genotypes. By contrast, CV2, which simulates incomplete field trials, maintained robust accuracy, highlighting the potential for GS to reduce phenotyping intensity without substantial loss of information. This finding is consistent with studies in wheat and barley, where sparse multi-environment phenotyping has been shown to be sufficient when combined with GS (Rutkoski et al., 2014). Finally, CV0, which reflects predictions for new environments, produced the highest accuracies for SV and comparable accuracies for IR, emphasizing the stability of genetic effects across locations and years. The strong performance under CV0 likely reflects the moderate to high heritability observed in most environments and suggests that GS can reliably extrapolate SR resistance to novel trial sites. Similar conclusions have been drawn in wheat, where GS has shown strong potential for predicting disease resistance across diverse production environments (Juliana et al., 2017).

Together, these findings demonstrate that GS provides a powerful framework for improving stem rust resistance in oats. The consistent superiority of Bayesian and other linear models indicates that the underlying architecture is predominantly additive, while the cross-validation results confirm that GS can effectively reduce reliance on extensive, multi-environment phenotyping. Importantly, the comparable performance of the 3K and GBS platforms suggests that both marker systems provide adequate coverage for GS in this population, despite their contrasting marker distributions and LD properties. From a computational perspective, however, the 3K array offers clear advantages. With approximately six times fewer markers than the GBS dataset, the 3K panel reduces memory demands, file sizes, and computation time for model training and cross-validation without compromising predictive ability. This efficiency can translate into substantially faster analyses and lower computational costs, making the 3K panel particularly attractive for routine genomic prediction, while GBS may remain useful when higher marker density is needed for fine mapping or across-panel applications.

## 5 Conclusion

This study demonstrates the utility of genomic selection for improving stem rust resistance in oats by leveraging multi-environmental data from the SOAP. Across cross-validation scenarios, predictive ability was moderate to high, supported by the strong genetic signal and heritability of stem rust resistance traits. Bayesian models consistently achieved the highest accuracies, with GBLUP and RRBLUP performing nearly as well, while machine-learning approaches were less effective. These results suggest that the genetic architecture of resistance is largely additive and can be efficiently captured by linear models. Importantly, both the 3K SNP array and GBS platform provided sufficient marker coverage, despite differences in marker distribution and LD patterns, indicating flexibility in genotyping strategy for GS implementation. From a breeding perspective, the strong performance of GS under CV2 and CV0 highlights its potential to reduce reliance on exhaustive multi-environment phenotyping and to support the prediction of new genotypes and environments. Collectively, these findings validate GS as a powerful and practical tool for accelerating the development of stem rust–resistant oat cultivars and provide a framework for integrating genomic prediction into oat breeding pipelines.

## Data Availability

The datasets presented in this study can be found in online repositories. The names of the repository/repositories and accession number(s) can be found in the article/**Supplementary Material**. The link to the repository is https://github.com/janamacharya/Oat_SR_GS_data.
