# Supplementary material for: Genomic prediction for stem rust resistance in the southern United States elite oat (Avena sativa L.) germplasm
Source: Front Plant Sci. 2026 Mar 19;17:1795871. doi: 10.3389/fpls.2026.1795871 (PMC13044010; doi:10.3389/fpls.2026.1795871)
Supplement: Supplementary file 1 [file DataSheet1.docx]

**Table S1**: Descriptive statistics for stem rust traits at the adult stage, based on combined data from seven location–year (environment) combinations

| **Trait^a^** | **Min** | **Max** | **Mean** | **SD** | ***H^2^*** | ***p*-value^b^** |
| --- | --- | --- | --- | --- | --- | --- |
| SV_ | 0 | 90 | 72.49 | 21.67 | 0.87 | <0.0001 |
| IR | 0 | 1 | 0.93 | 0.16 | 0.81 | <0.0001 |

^a^Trait: IR, infection response; and SV, disease severity.

*^b^p*-value Significance of genotype effect; <0.001 indicates highly significant genetic variation across environments


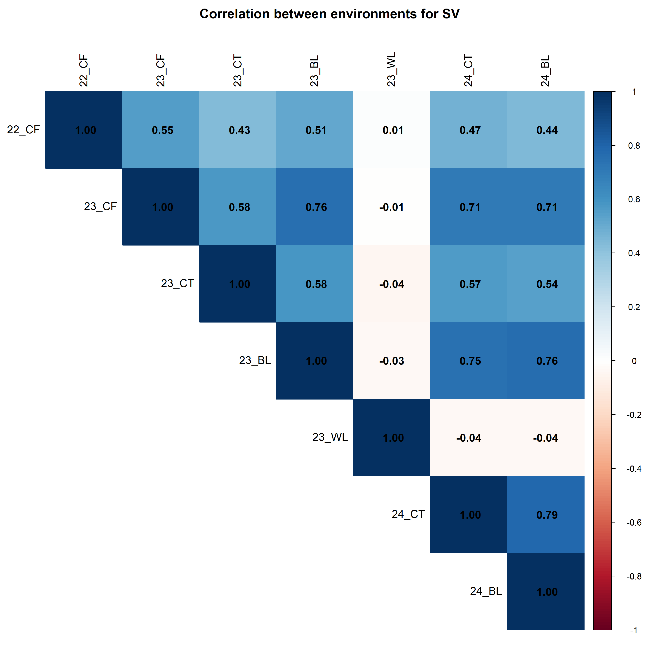


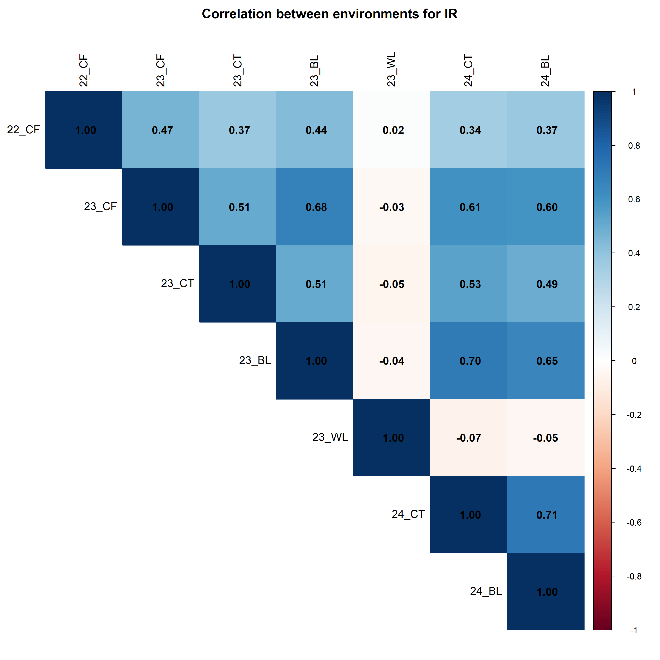


**Figure S1.** Pairwise Pearson correlations among seven location–year (environment) for stem rust traits evaluated at the adult plant stage: (a) infection response (IR) and (b) severity (SV).


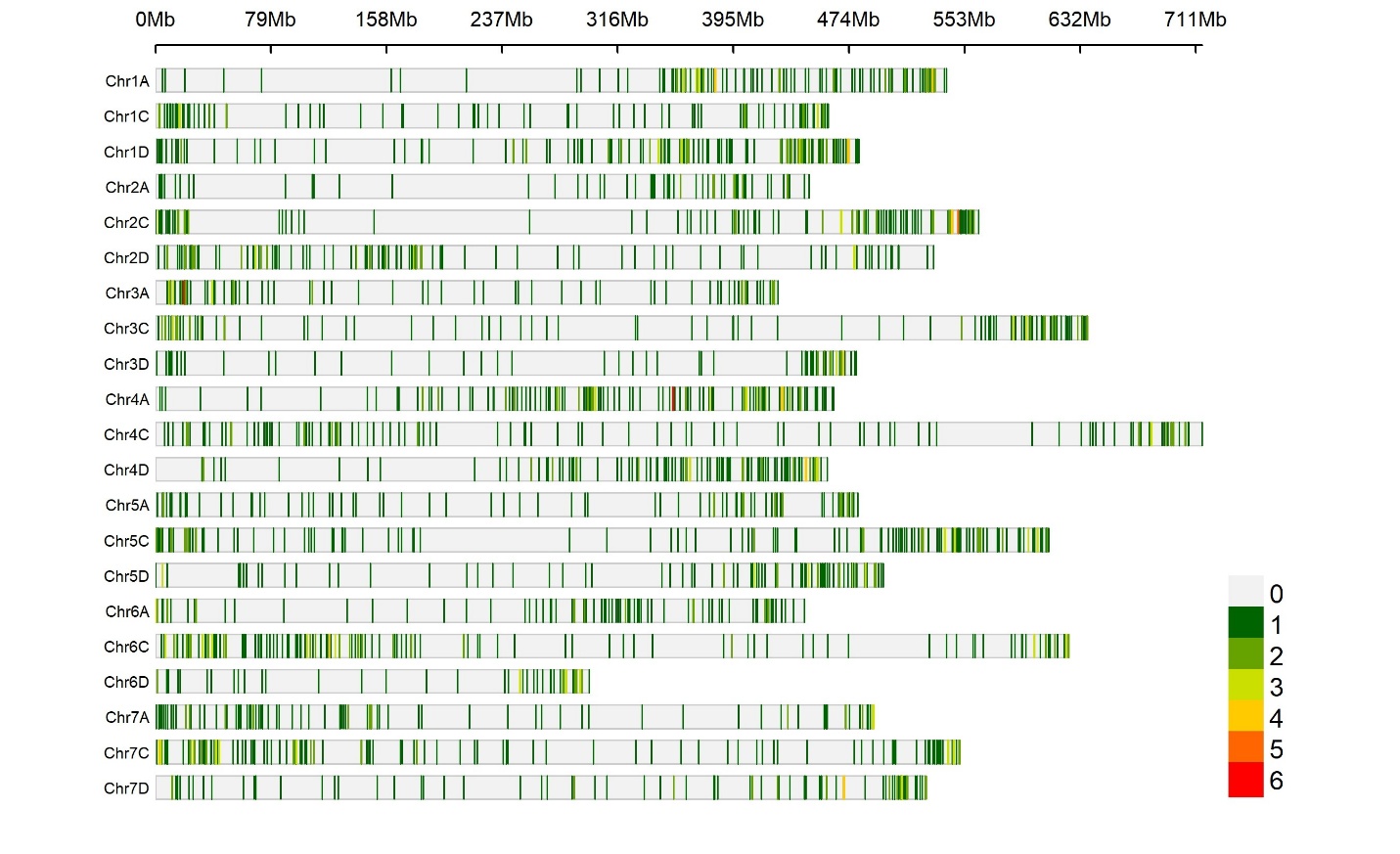


**Figure S2:** A genome-wide SNP density plot was generated using 2134 SNP markers using 3K SNP data, with the horizontal axis representing chromosome length (Mb). Different colors indicate variations in SNP density distribution across chromosomes. The number of SNPs identified on chromosomes 1A, 1C, 1D, 2A, 2C, 2D, 3A, 3C, 3D, 4A, 4C, 4D, 5A, 5C, 5D, 6A, 6C, 6D, 7A, 7C, and 7D is 110, 89, 138, 56, 117, 113, 86, 106, 61, 139,115, 111, 80, 133, 91, 86, 151, 49, 90, 129, and 84, respectively


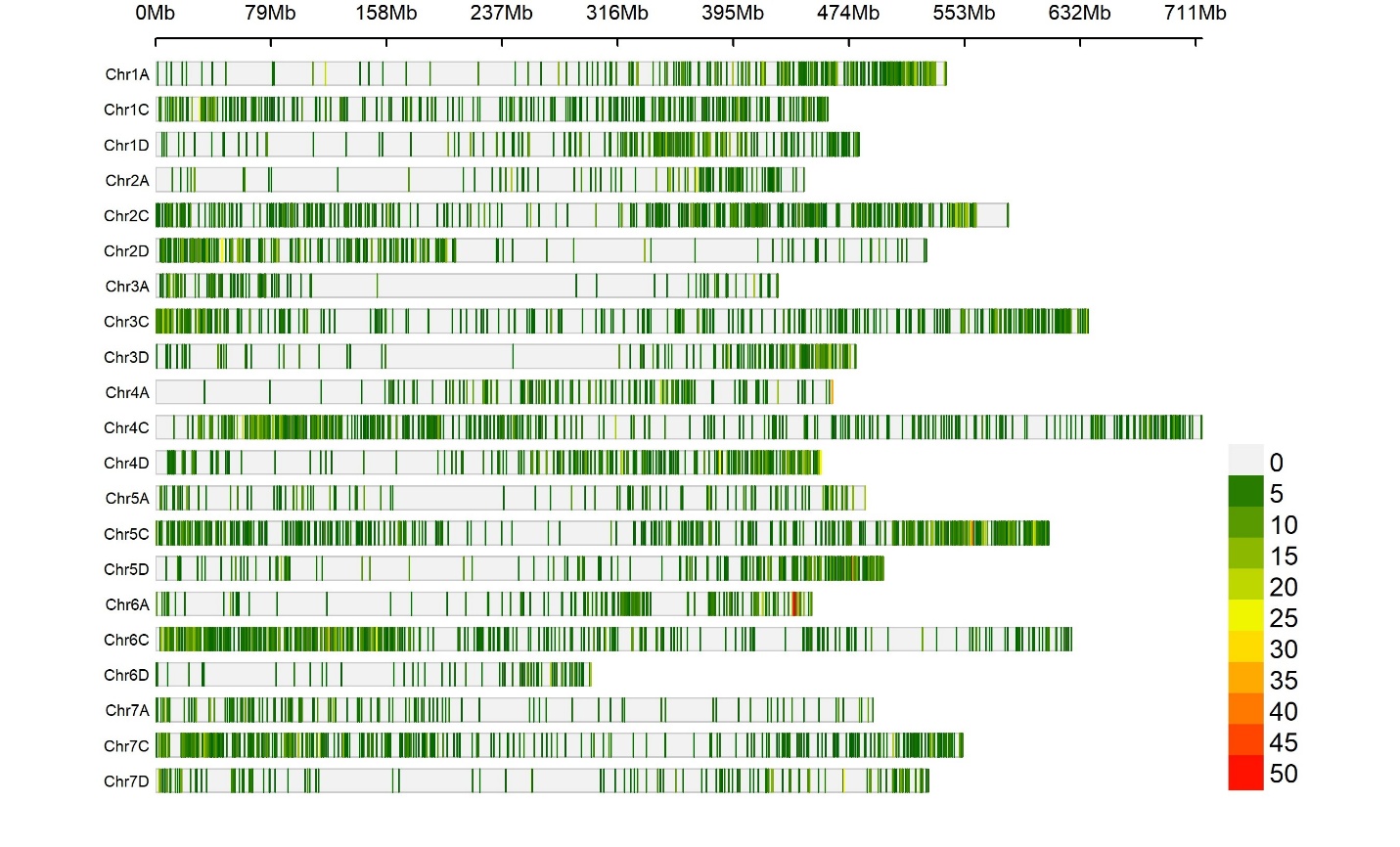


**Figure S3:** A genome-wide SNP density plot was generated using 12,914 SNP markers using genotyping by sequencing (GBS) data, with the horizontal axis representing chromosome length (Mb). Different colors indicate variations in SNP density distribution across chromosomes. The number of SNPs identified on chromosomes 1A, 1C, 1D, 2A, 2C, 2D, 3A, 3C, 3D, 4A, 4C, 4D, 5A, 5C, 5D, 6A, 6C, 6D, 7A, 7C, and 7D is 751, 646, 580, 317, 903, 638, 234, 774, 450, 418,1040, 736, 346, 1091, 567, 525, 1084, 158, 358, 878, and 419, respectively
